# Vitamin B_6_ is governed by the local compartmentalization of metabolic enzymes during growth

**DOI:** 10.1126/sciadv.adi2232

**Published:** 2023-09-08

**Authors:** Carolina N. Franco, Laurence J. Seabrook, Steven T. Nguyen, Ying Yang, Melissa Campos, Qi Fan, Andrew C. Cicchetto, Mei Kong, Heather R. Christofk, Lauren V. Albrecht

**Affiliations:** ^1^ Department of Pharmaceutical Sciences, School of Pharmacy & Pharmaceutical Sciences, University of California, Irvine, Irvine, CA, USA.; ^2^ Department of Developmental and Cell Biology, School of Biological Sciences, University of California, Irvine, Irvine, CA, USA.; ^3^ Department of Molecular Biology and Biochemistry, University of California, Irvine, Irvine, CA, USA.; ^4^ Department of Biological Chemistry, David Geffen School of Medicine, University of California, Los Angeles, Los Angeles, CA, USA.

## Abstract

Vitamin B_6_ is a vital micronutrient across cell types and tissues, and dysregulated B_6_ levels contribute to human disease. Despite its importance, how B_6_ vitamer levels are regulated is not well understood. Here, we provide evidence that B_6_ dynamics are rapidly tuned by precise compartmentation of pyridoxal kinase (PDXK), the rate-limiting B_6_ enzyme. We show that canonical Wnt rapidly led to the accumulation of inactive B_6_ by shunting cytosolic PDXK into lysosomes. PDXK was modified with methyl-arginine Degron (MrDegron), a protein tag for lysosomes, which enabled delivery via microautophagy. Hyperactive lysosomes resulted in the continuous degradation of PDXK and B_6_ deficiency that promoted proliferation in Wnt-driven colorectal cancer (CRC) cells. Pharmacological or genetic disruption of the coordinated MrDegron proteolytic pathway was sufficient to reduce CRC survival in cells and organoid models. In sum, this work contributes to the repertoire of micronutrient-regulated processes that enable cancer cell growth and provides insight into the functional impact of B_6_ deficiencies for survival.

## Teaser

Lysosomes link Wnt and vitamin B6 metabolism by degrading the rate-limiting B6 enzyme during cell growth and in cancer.

## INTRODUCTION

**Fig. 1.**
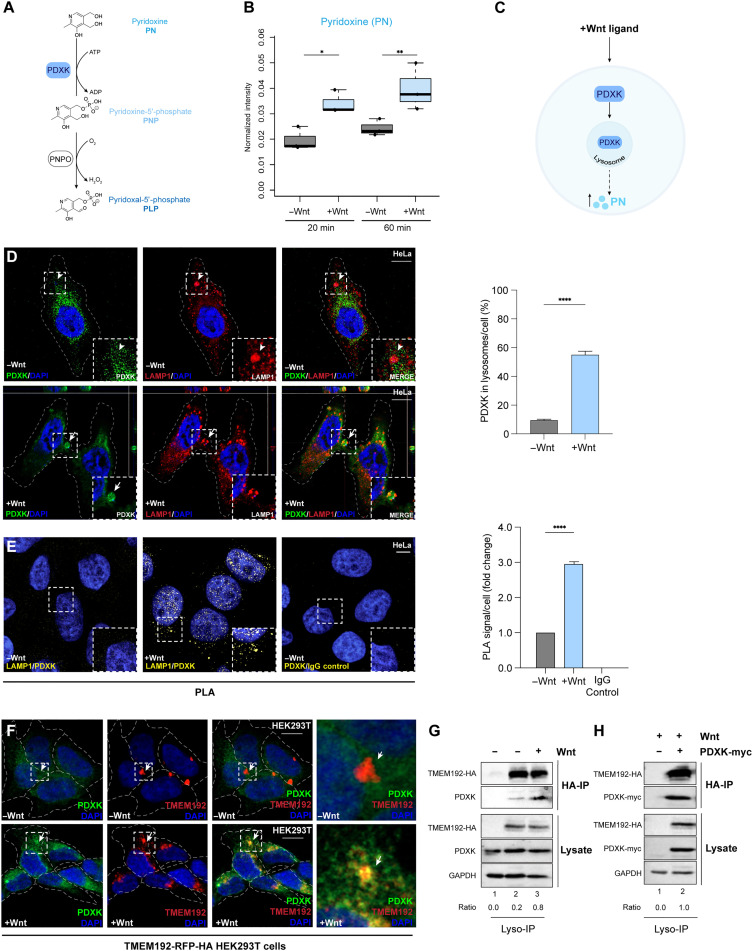
Wnt orchestrates vitamin B_6_ through protein compartmentalization. (**A**) Inactive B_6_, pyridoxine (PN), is phosphorylated by pyridoxal kinase (PDXK) to generate pyridoxine-5′-phosphate (PNP) and oxidized by pyridoxine 5′-phosphate oxidase (PNPO) to produce pyridoxal 5′-phosphate (PLP), the metabolically active B_6_. (**B**) Intracellular PN in HeLa cells following Wnt (blue) or control (gray) treatments (20 or 60 min). (**C**) Scheme depicting Wnt-induced accumulation of PN via PDXK sequestration into lysosomes. (**D**) PDXK (green) and LAMP1 (red) colocalization during Wnt or control treatments (20 min) in HeLa cells assessed by IF analyses (scale bar, 10 μm; 63× magnification). Arrowheads and arrows indicate cytosolic and lysosomal PDXK, respectively. Graph depicts quantification of PDXK-positive lysosomes (LAMP1) using IF images from more than 10 fields (*n* < 20). (**E**) PDXK and LAMP1 interactions with Wnt or control treatments (20 min) in HeLa cells assessed by PLA. PDXK and mouse IgG antibody pairs were used as a negative control. PLA signals indicate protein-protein interactions within 40 to 100 nm and were pseudo-colored yellow (scale bar, 10 μm; 40× magnification). Graph depicts quantification of PLA signals using FIJI particle analysis using images from over 10 fields (*n* < 30). (**F**) PDXK (green) and TMEM192-RFP colocalization during Wnt or control treatments (20 min) in HEK293Ts (scale bar, 10 μm; 20× magnification). (**G**) Lyso-IP using HA-TMEM192 during Wnt or control treatments (20 min) from HEK293T assessed by immunoblotting (IB). Ratios indicate immunoprecipitated endogenous PDXK relative to input (lysate). Nontransfected HEK293T cells were used a control. (**H**) Lyso-IP using HA-TMEM192 during Wnt or control treatments in PDXK transfected HEK293Ts. Ratios indicate immunoprecipitated PDXK in each condition relative to input. (G) and (H) are biological duplicates. All other data represent biological triplicates. *P < 0.05; **P < 0.01; ****P < 0.0001. Student’s *t* test; means ± SEM. See also fig. S1.

B vitamins are essential micronutrients that enable fundamental processes in metabolism and cell signaling (*1*). Of the eight different B vitamins, intensive research efforts have been devoted to vitamins B_9_ (folate) and B_12_ (cobalamin), whose deficiencies lead to birth defects and blood diseases, respectively (*2*). Once considered, the forgotten B vitamin, B_6_, has emerged to be a key regulator of amino acid, nucleic acid, and lipid metabolism (*3*). The active form of vitamin B_6_, pyridoxal 5′-phosphate (PLP), serves as a cofactor for more than 160 enzymes (*3*). Vitamin B_6_ is obtained from diet, and pyridoxal kinase (PDXK) is the enzyme responsible for converting inactive B_6_ into PLP across cell types and tissues (Fig. 1A) (*4*–*6*). The dysregulation of vitamin B_6_ metabolism contributes to several human diseases. Genetic mutations in *PDXK *have been identified in polyneuropathy (*7*) and type 2 diabetes (*8*, *9*). Vitamin B_6_ deficiencies have been reported across numerous cancers, and restoring B_6_ levels has been shown to be protective in the case of lung, breast, and colon (*10*). How vitamin B_6_ levels are regulated across tissues is not well understood. The low levels of B_6_ in cancer hint at a role for PDXK; however, the presence of *PDXK *mutations in cancer is less defined (*11*, *12*). Whether the dysregulation of PDXK may occur at the protein level is unknown, and the cellular pathways responsible for PDXK degradation have yet to be examined.

Cellular metabolism requires precise integration of biological time frames. For instance, fluctuations of metabolite concentrations occur within seconds, while the downstream cellular responses within tissues are relayed after hours (*13*, *14*). How the timing of metabolic and gene networks are integrated in cells is an active area of research. In the case of signal transduction events, specificity is endowed by the rapid organization of proteins into distinct compartments, which allows cells to precisely tune the timing of biochemical activities (*15*, *16*). There has been a recent growing appreciation that cells may similarly use the separation of cellular domains to achieve spatial and temporal control of metabolism (*17*). Elucidating the mechanisms through which distinct subcellular remodeling promotes metabolic pathways across different cell types and tissues could have value for developing interventions for cancer cell metabolism. The metabolic pathways that exploit such spatial compartmentalization are still being uncovered. Canonical Wnt signaling is an essential pathway that uses several forms of subcellular compartmentalization to promote diverse biological processes with a common core set of protein players (*18*). Further, Wnt signaling has also emerged to be an important regulator of cellular metabolism (*19*–*21*). Whether Wnt uses subcellular compartmentation of metabolic enzymes to orchestrate metabolism remains to be defined and offered an ideal system for further investigation.

β-Catenin lies at the heart of the canonical Wnt pathway where cytosolic β-catenin is continually degraded by the destruction complex, which is composed of adenomatous polyposis coli (APC), glycogen synthase kinase 3 (GSK3), casein kinase 1, Disheveled, and Axin (*18*, *22*). Upon Wnt activity, β-catenin is stabilized and initiates β-catenin–dependent transcription of the lymphoid enhancer-binding factor/T cell factors (*23*). Secreted Wnt ligands bind to the extracellular domains of receptors, low-density receptor protein 6, and Frizzled and initiate several distinct cytosolic organization processes that allow for β-catenin accumulation (*24*–*27*). Examples of such subcellular architectural remodeling include the assembly of cytosolic destruction complexes into signalosomes (*27*, *28*), phase condensates (*29*), or endolysosomes (*30*–*32*). Beyond the destruction complex, Wnt ligands also induce the rapid remodeling of many cytosolic proteins and the abundance of several intracellular metabolites, such as glucose and amino acids, within minutes (*33*). Whether Wnt signaling uses subcellular compartmentalization for the regulation of cellular metabolism has not been previously explored.

Colorectal cancer (CRC) is the second leading cause of cancer-associated deaths (1 million cases in the United States) (*18*, *34*–*36*). The most common genetic mutations in CRCs occur in the Wnt pathway (*34*, *37*). Mutational signatures across CRCs have been well documented, yet treatment options remain limited as the molecular mechanisms underlying tumorigenic progressions are incompletely understood. Aberrant Wnt signaling has been attributed to several metabolic pathways associated with CRC progression including Warburg metabolism, glutamine metabolism, serine-glycine metabolism, and one-carbon metabolism (*19*–*21*, *33*, *38*, *39*). Vitamin B_6_ deficiencies have been reported across clinical cohorts of CRCs (*40*–*51*). Eighty percent of circulating vitamin B_6_ is absorbed from food in the intestinal microflora (*11*). Whether Wnt signaling regulates B_6_ metabolism and plays a role in cancer cell progression has yet to be studied.

The present study describes investigations of the interplay between Wnt signaling and vitamin B_6_ metabolism. Previous studies have established that Wnt shuttles cytosolic proteins into lysosomes to propagate a variety of cellular behaviors (*30*, *31*, *33*, *38*, *52*–*54*). Here, we show that Wnt similarly exploits lysosomes to shift the availability and abundance of intracellular B_6_ metabolite levels through a methylation-dependent process. These studies implicate key roles for vitamin B_6_ in the processes of cell growth and proliferation and hint at a therapeutic vulnerability to exploit in CRCs.

## RESULTS

### Wnt signaling regulates vitamin B_6_ metabolism

Canonical Wnt regulates metabolism in processes of development, stem cell maintenance, and tumorigenesis (*18*). Such regulation occurs rapidly as stimulation of the Wnt pathway increases the levels of intracellular amino acids and glucose after only minutes of Wnt ligand addition (*19*, *33*). The central role of Wnt signaling for metabolism and in CRCs provided a premise for the present study focused on vitamin B_6_, a micronutrient that is deficient in patients with CRC and associates with worse prognosis and survival (*40*–*51*).

To first test whether Wnt directly affects cellular B_6_ levels, we applied a metabolomics-based approach using liquid chromatography–mass spectrometry (LC-MS). For this assay, cells were first treated with inhibitor of Wnt production 2 (IWP-2) to block endogenous Wnt secretion (*55*) and subsequently with Wnt ligands or control buffer for time points of 20 and 60 min, as in previous metabolomic analyses (*33*). Cell pellets were extracted with trichloroacetic acid and analyzed by LC-MS. Compared to control conditions, Wnt led to an increase in the levels of pyridoxine (PN), the inactive form of vitamin B_6_, after only 20 min, and PN continued to accumulate after 60 min (Fig. 1B). High PN likely indicates low PLP. Thus, Wnt signaling altered vitamin B_6_ metabolite levels on a minute time scale.

We next examined the molecular basis through which Wnt ligands increased intracellular PN levels. Previous work demonstrates that Wnt stimulation rapidly remodels protein distributions in the cytosol, which drives the spatiotemporal regulation of protein activities to promote downstream signaling events. For instance, Wnt ligands redirect cytosolic GSK3 away from its substrate, β-catenin, within 20 min of pathway activation (*30*, *33*). The time frame that Wnt increased intracellular PN after 20 and 60 min led us to test whether Wnt may similarly segregate the subcellular location of B_6_ enzymes to regulate the dynamics of B_6_ metabolism (Fig. 1C).

### Wnt signaling recompartmentalizes vitamin B_6_ enzymes

PDXK is the cytosolic enzyme responsible for converting inactive B_6_ (PN) into its active form, PLP (Fig. 1A). Given the established kinetics of PDXK enzymatic activity (*8*), we extrapolated that the observed accumulation of PN after an hour of Wnt treatment could result from the reorganization of PDXK within the window of 5 to 20 min. To test this hypothesis, PDXK was visualized in cells using immunofluorescence (IF) analyses. In steady-state cells, endogenous PDXK was localized in the cytoplasm, as expected (Fig. 1D, arrowheads). In contrast, Wnt3a dramatically shifted cytosolic PDXK into tightly organized punctate structures after 20 min (Fig. 1E and fig. S1A). To characterize punctate PDXK, lysosomal-associated membrane protein 1 (LAMP1) colocalization analyses were performed. Compared to control cells, PDXK was colocalized with LAMP1 by Wnt (Fig. 1D). Protease protection assays were also performed as a complementary approach to evaluate vesicular localization (*30*). For this assay, live cells are treated with proteinase K and digitonin, which disrupt cholesterol patches in the plasma membrane and leave intracellular vesicular structures intact (fig. S1B). PDXK was protected in membrane-bounded organelles only in the presence of Wnt activation by IF analyses (fig. S1C). Triton X-100 permeabilization of all membranes released PDXK from protected structures as a positive control (fig. S1C). These data support a model whereby Wnt signaling led to the compartmentalization of PDXK into lysosomes to rapidly increase the inactive B_6_ vitamer, PN.

To further evaluate the lysosomal delivery of PDXK, we applied an orthogonal approach to evaluate protein-protein interactions within native cellular structures using proximity ligation assay (PLA) (*56*). For this assay, oligonucleotide-tethered secondary antibodies generate signals via polymerase-driven rolling circle amplification that incorporates fluorescently labeled nucleotides when two protein targets are within 40 to 100 nm. We used a LAMP1 antibody that recognizes the luminal side of the protein to help provide insight into endogenous PDXK within lysosomes in intact cells during Wnt treatments. PLA signals were not observed with antibody pairs between PDXK and mouse immunoglobulin G (IgG) that were used as a negative control (Fig. 1E). In contrast, PLA signals were found with PDXK and LAMP1 antibody pairs, which were increased during Wnt treatments compared to control-treated cells (Fig. 1E).

Last, we performed a biochemical approach to detect PDXK in purified lysosomes from cells. Traditional density-based centrifugation approaches to isolate lysosomes can obscure detection of less abundant proteins. To avoid this, we used a lysosome-immunoprecipitation (Lyso-IP) method that allows for the rapid isolation of pure, intact lysosomes from live cells (*57*). Human embryonic kidney (HEK) 293T cells stably expressing a lysosomal membrane protein, transmembrane protein 192 (TMEM192), fused to the human influenza hemagglutinin (HA), were used to isolate lysosomes with HA-magnetic beads, as previously described (*57*, *58*). Nontransfected HEK293T cells were used as a control. IF analyses confirmed that PDXK was colocalized with lysosomal protein TMEM192 in HEK293T cells following Wnt treatments of 20 min (Fig. 1F). Next, Lyso-IP was performed with HA-magnetic beads on cells treated with Wnt or control before cell lysis. Lysates confirmed that endogenous PDXK was found in the stable cell line and in nontransfected HEK293T cells by immunoblot analyses. TMEM192-HA expression was restricted to HA-Lyso cells, as expected (Fig. 1G). TMEM192-HA was constant in immunoprecipitated lanes, while PDXK was enriched in lysosomes during Wnt treatments compared to controls. To further validate these findings, an exogenous myc-tagged PDXK fusion construct was transfected into Lyso-IP cells, and HA IP was performed following Wnt treatments (Fig. 1H). PDXK-myc was similarly enriched in purified lysosomes, as seen with endogenous PDXK (Fig. 1H). These data further support that PDXK becomes spatially restricted into lysosomes, which shifted intracellular B_6_ concentrations to PN, the inactive form.

### Vitamin B_6_ enzymes are degraded in lysosomes

**Fig. 2.**
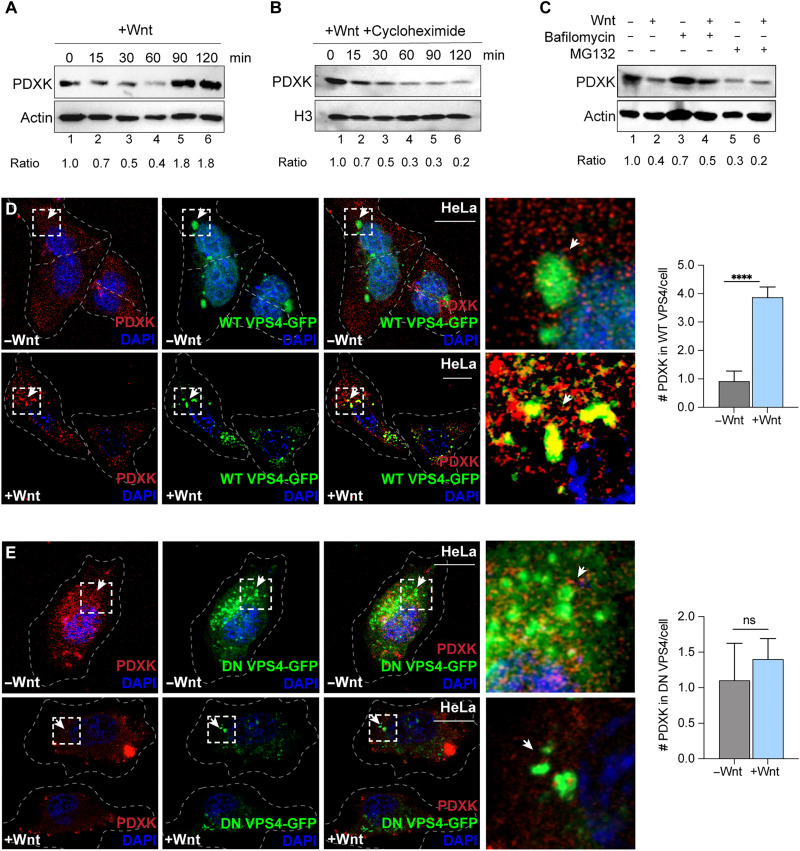
PDXK is degraded in lysosomes during Wnt signaling. (**A**) PDXK protein levels in HeLa lysates during a Wnt time course by IBs. Ratios indicate PDXK relative to loading control. (**B**) PDXK protein levels in HeLa lysates during a Wnt time course in the presence of cycloheximide (10 μM) by IBs. Ratios indicate PDXK relative to loading control. (**C**) PDXK protein levels in HeLa cells treated with lysosome inhibitor, bafilomycin (200 nM), or proteasome inhibitor (5 μM) with Wnt or control treatments (60 min) by IBs. Ratios indicate PDXK relative to loading control. (**D** and **E**) Endogenous PDXK (red) staining with WT or DN VPS4-GFP (green) in HeLa cells with control or Wnt treatments (20 min) by IF. Graphs indicate PDXK colocalization with WT VPS4-GFP (arrows). PDXK localization was decreased in DN VPS4-GFP-positive lysosomes (scale bar, 10 μm; 20× magnification). Quantification was performed using 10 fields (*n* < 10). All data represent biological triplicates. ****P < 0.0001. ns., not significant. Student’s *t* test; means ± SEM. See also fig. S1.

Proteins are degraded through two distinct cellular pathways: proteasomes or lysosomes. Short-lived proteins are targeted to proteasomes, while lysosomes degrade macromolecules in addition to other types of cellular material (*59*). While PDXK is a clear proteasomal target based on its small size and cytosolic location, the notable localization of PDXK with LAMP1 suggested otherwise. We asked whether PDXK was redirected into vesicular structures for temporary storage or whether PDXK was degraded. To distinguish between these two possibilities, we evaluated protein levels over a Wnt time course. After only 15 min with Wnt, PDXK was decreased, and protein levels steadily diminished with maximal degradation after an hour, as detected by immunoblotting (Fig. 2A, compare lanes 1 and 2). Intriguingly, PDXK restabilized after 90 min, which suggested that protein synthesis was induced. To test this, cycloheximide assays were performed and showed that a Wnt time course similarly reduced PDXK after 90 min when protein synthesis was blocked (Fig. 2B). Epidermal growth factor–stimulated cells had no effect on PDXK protein levels, indicating that catabolism was specific to Wnt (fig. S1D). Thus, the accumulation of PN after an hour of Wnt treatment coincided with the time frame of PDXK degradation.

To parse out the relative contribution of distinct proteolytic pathways on PDXK, protein levels were evaluated with inhibitors of the lysosome and the proteasome. Lysosomal inhibitor, bafilomycin, was sufficient to stabilize PDXK levels in the presence of Wnt signaling (Fig. 2C, compare lanes 3 and 4). In contrast, treatment with the proteasomal inhibitor, MG132, had no effect on PDXK levels with or without Wnt (Fig. 2C, compare lanes 5 and 6). These data suggest that PDXK degradation predominantly occurs in lysosomes during Wnt signaling.

### Cytosolic delivery into lysosomes occurs via microautophagy

We next examined the molecular machinery responsible for the lysosomal delivery of PDXK. Three predominant forms of autophagy deliver cellular content into lysosomes: (macro)autophagy (*59*), microautophagy (*60*), and chaperone-mediated autophagy (CMA) (*61*). To examine potential delivery modes for PDXK, we performed sequence analyses and found that PDXK did not have classic CMA motifs (KFERQ). This led us to evaluate the roles of additional regulatory pathways that may contribute to PDXK delivery. Previous work illustrates that Wnt drives cytosolic proteins such as GSK3 into lysosomes via microautophagy and Endosomal Sorting Complex Required for Transport (ESCRT) machinery (*30*–*32*, *54*). Vacuolar protein sorting 4 (VPS4) is an adenosine triphosphatase (ATPase) that recognizes ESCRT-III to facilitate invaginations into the limiting membrane of endosomes and multivesicular bodies (*60*). VPS4–green fluorescent protein (GFP) was exploited as a tool to examine microautophagy in our system by comparing wild-type (WT) with a dominant-negative (DN) VPS4-GFP point mutant that lacks ATPase activity (*30*, *31*). Wnt ligands rapidly induced the colocalization of WT VPS4-GFP with endogenous PDXK (Fig. 2D). In contrast, Wnt had no effect on PDXK colocalization with DN VPS4-GFP (Fig. 2E). Additional studies were performed to include a triple stain with LAMP1. These analyses further show a colocalization of PDXK with lysosomes that contained WT VPS4-GFP, while DN VPS4-GFP lacked PDXK staining (fig. S, 1E and F). We conclude that VPS4 ATPase activity promoted the microautophagic entry of PDXK into lysosomes.

### Protein methylation drives cytoplasmic delivery into lysosomes

Protein methylation is a posttranslational modification that has emerged to be a regulator of protein degradation in proteasomes (*62*, *63*) and lysosomes (*30*). Proteasomal degradation via lysine methylation events are known as methyl degrons and are catalyzed by protein lysine methyltransferases (*62*). In contrast, arginine methylation leads to protein degradation in lysosomes where modifications are catalyzed by protein arginine methyltransferases (PRMTs) (*30*). To distinguish between these two methylation-dependent degradation processes, the term methyl arginine degron, or MrDegron, was coined and specifically refers to arginine methylation events on substrates that are degraded in lysosomes.

**Fig. 3.**
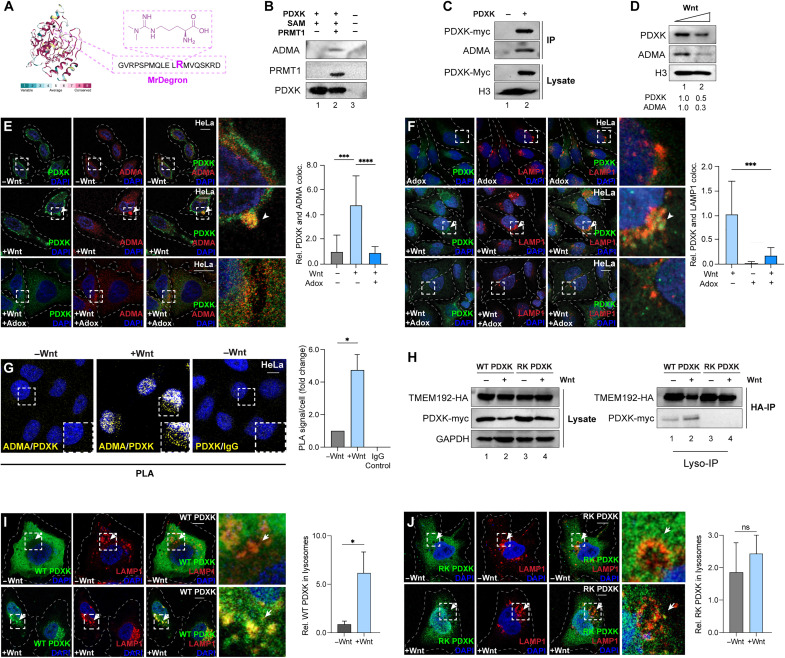
Protein methylation via MrDegron triggers cytolysosomal shuttling of PDXK. (**A**) PDXK sequence conservation using Consurf; maroon and cyan indicate high and low conservation, respectively. Methyl arginine Degron (MrDegron) indicated in pink at a conserved methylated arginine within an IDR. (**B**) In vitro methylation assay using recombinant His-PDXK and SAM incubated with or without GST-PRMT1 assessed by IB using PDXK, PRMT1, and ADMA (asymmetric dimethyl arginine) antibodies. (**C**) IP of *myc-PDXK*–transfected HEK293Ts assessed by IB. (**D**) Endogenous PDXK and ADMA during Wnt treatments. PDXK degradation by Wnt coincides with lower ADMA levels from the degradation of methylated proteins. Ratios indicate PDXK relative to loading control. (**E** and **F**) PDXK (green) with ADMA and LAMP1 (red) during treatments with Wnt, Adox, or DMSO in HeLa cells by IF (scale bar, 10 μm; 20×). Arrowheads indicate colocalization. Quantification of PDXK colocalization with ADMA or LAMP1 using IF images from over 10 fields (*n* < 40). (**G**) PDXK and ADMA with Wnt or control treatments (20 min) in HeLa cells assessed by PLA (yellow). PDXK and mouse IgG antibody pairs were used as a negative control (scale bar, 10 μm; 20×). Quantification of PLA signals with images from over 10 fields (*n* < 30). (**H**) Lyso-IP using HA-TMEM192 HEK293Ts transfected with WT or RK *PDXK *during Wnt or control treatments. (**I** and **J**) WT and RK PDXK (green) with LAMP1 (red) in Wnt or control treatments by IF (scale bar, 10 μm; 63×). Arrows and arrowheads mark lysosomal WT PDXK and cytosolic RK, respectively. Quantification of PDXK-positive lysosomes over 10 fields (*n* < 20). (C) and (D) are biological duplicates. All other data are biological triplicates. *P < 0.05; ***P < 0.001; ****P < 0.0001. Student’s *t* test; means ± SEM. See also figs. S2 and S3.

Type I and type II PRMTs catalyze asymmetric (ADMA) and symmetric (SDMA) di-methylation on arginine residues, respectively (*64*). MrDegron modifications occur as type I ADMA modifications where the modified arginine is frequently within intrinsically disordered regions (IDRs) and often have negatively charged residues or serine residues nearby. Similar to previously identified MrDegron targets, PDXK contains a putative methylation site on a conserved arginine residue within an IDR containing serine residues and negatively charged residues (Fig. 3A). MrDegron events are known to use microautophagy for lysosomal delivery (*30*). With the present data that PDXK enters lysosomes via microautophagy, we reasoned that PDXK may be an MrDegron target.

To assess whether PDXK is an MrDegron target, we examined whether PDXK was modified by arginine methylation. PRMT1 is responsible for 85% of type I ADMA modifications (*64*, *65*) and is the primary methyltransferase for lysosomal degradation (*30*, *38*). Cell-free in vitro assays were performed using recombinant human glutathione *S*-transferase (GST)–PRMT1 and His-PDXK proteins that were combined with the methyl cofactor *S*-adenosyl methionine (SAM). Immunoblotting was performed using an ADMA-specific antibody, which showed that arginine methylation was observed with His-PDXK in the presence of SAM compared to the negative control (Fig. 3B). To further evaluate PDXK methylation in cells, PDXK-Flag-myc was transfected into HEK293T cells and subjected to Flag-IPs. Consistent with the in vitro methylation analyses, purified PDXK-Flag-myc was recognized by ADMA antibodies in immunoblotting assays (Fig. 3C). Similarly, endogenous PDXK was also recognized by ADMA in cell lysates collected during Wnt treatments (Fig. 3D). The reduced levels of ADMA and PDXK after 1 hour of Wnt treatment further support evidence that PDXK was degraded. Last, we aimed to identify the precise residue that was modified by PRMT1 using MS. PDXK and PRMT1 in vitro reactions were compared with or without SAM, and PRMT1 was subsequently removed by GST purification. Compared to the minus SAM controls, PDXK was modified by di-methylation at arginine-292 (R292), which is highly conserved and falls within the putative MrDegron residue in the C-terminal IDR (Fig. 3A and fig. S2). These data support that PDXK is an MrDegron substrate.

### MrDegron drives PDXK shuttling into lysosomes

Previous studies demonstrate that lysosomal delivery of methylated proteins can be monitored using ADMA antibodies (*30*, *38*). Cells were treated with Wnt or control buffer and costained with PDXK and ADMA antibodies (Fig. 3E). In control cells, ADMA was diffuse across the cytoplasm, as previously reported (*30*). Upon 20-min Wnt treatments, punctate ADMA structures formed, consistent with previous studies (*30*, *38*). Similarly, Wnt treatments were sufficient to shift endogenous PDXK into punctate structures that were colocalized with ADMA-positive structures by IF analyses (Fig. 3E). Further, Wnt treatments also led to increased colocalization of PDXK with PRMT1, compared to control IWP-2 alone (fig. S3A). To further explore the relevance of PRMT activity, analyses were performed with adenosine dialdehyde (Adox), an inhibitor of protein arginine methylation (*64*). Adox-treated cells dramatically decreased PDXK colocalization with ADMA (Fig. 3E) and PRMT1 (fig. S3B) in Wnt-treated cells. Further, Adox was similarly sufficient to reduce PDXK trafficking into lysosomes by Wnt (Fig. 3F). Together, these IF analyses support a mode whereby PDXK becomes modified by arginine methylation during Wnt signaling. To further examine endogenous PDXK methylation status in cells during Wnt treatments, we applied PLA analyses to evaluate protein posttranslational modification states in native cells. Cells were incubated with antibodies toward PDXK, ADMA, or mouse IgG as a negative control. While only few PLA signals were generated in the control IWP-2 treatments, Wnt stimulation increased PLA signals between PDXK and ADMA pairs after 20 min (Fig. 3G). PLA signals were not observed in the control antibody incubations with PDXK and mouse IgG antibodies (Fig. 3G). These data provide a model whereby active PRMT1 was recruited to PDXK and initiates MrDegron modifications for PDXK delivery into lysosomes.

### MrDegron mutations disrupt lysosomal delivery

To functionally evaluate the loss of PDXK MrDegron, we applied a genetic approach using site-directed mutagenesis to generate a methyl-deficient mutant of PDXK (Fig. 3H). R292 was selected for these analyses as it falls within an unstructured IDR region and was identified by our MS analyses of purified PDXK protein. R292 was mutated into a lysine (RK) that maintains the charge state while disrupting PRMT-mediated methylation (*30*). WT and RK PDXK were evaluated biochemically in lysates by immunoblotting (Fig. 3H). WT PDXK was decreased following 1 hour of Wnt treatments compared to IWP-2 control cells (Fig. 3H). In contrast, RK PDXK levels remained constant in both Wnt and IWP-2 treatments. Next, transfected Lyso-IP cells were used to purify lysosomes and demonstrated that WT PDXK was enriched in lysosomes with increased levels in the Wnt-treated condition (Fig. 3H). In contrast, RK PDXK was absent from the purified lysosomes despite a consistent expression in lysates. These data suggest that methylation at R292 is required for lysosomal delivery.

To evaluate entry into lysosomes, PDXK trafficking was monitored during Wnt stimulation. In steady-state conditions, both WT and RK PDXK (green) were cytosolic, as seen with endogenous PDXK (Fig. 3, I and J). Wnt rapidly increased the punctate appearance of WT PDXK and increased colocalization with lysosomes marked by LAMP1 (Fig. 3I). In contrast, RK PDXK was largely unchanged by Wnt treatments (Fig. 3J). Thus, these data support the Lyso-IP analyses and provide further confirmation that mutating R292 disrupted PDXK delivery into lysosomes. Together, these results showing that arginine methylation occurs on PDXK and plays a key role in lysosomal entry support the model that PDXK is an MrDegron substrate.

### Lysosomal inhibition restores active B_6_ and reduces cancer cell proliferation

CRC represents the second-leading cause of cancer-related deaths (*36*). The genetic alterations in CRC are well recognized (*34*) where the most frequent type of mutation occurs in the Wnt pathway (*18*, *34*–*36*). However, therapies are limited by a lack of knowledge of disease progression beyond the mutational profiles. The rewiring of cancer cell metabolism has been reported and could play a central role in tumorigenesis. Clinical studies have reported vitamin B_6_ deficiencies across CRC, despite an absence of *PDXK *genetic mutations (*40*–*51*). These studies hint that the dysregulation of PDXK may occur at the protein level in the cases where B_6_ deficiencies have been reported; however, this has never been previously studied. Our data show that PDXK protein levels were regulated by lysosomes, methylation, and Wnt signaling. In addition to hyperactive Wnt activity, CRCs have been shown to have overexpressed *PRMT1 *in mouse models and patient tissues (*66*). Aberrant Wnt activity increases lysosomal catabolism in CRC cells (*52*). Given this, we posited that intestinal tissues and CRC could offer an approach to study the role of PDXK MrDegron in regulating the dynamics of B_6_ metabolism.

We first examined whether hyperactive Wnt may contribute to vitamin B_6_ deficiencies via PDXK dysregulation in CRC. Toward this goal, SW480 CRC cells were selected as they contain* Apc* loss-of-function mutations, hyperactive lysosomal activity, and elevated *PRMT1 *expression (*37*, *52*, *66*). SW480s were examined by IF analyses, and PDXK colocalization was performed with ADMA, LAMP1, and PRMT1. SW480 cells showed colocalization of PDXK with ADMA (fig. S4A) and PRMT1 in punctate structures, even in the absence of Wnt ligands (fig. S4B). Further, PDXK was also highly enriched in lysosomal structures (fig. S4C). SW480 cell lysates had lower levels than in HEK293T or HeLa lysates (fig. S4D). Next, to test whether methyltransferase activity contributed to PDXK trafficking into lysosomes, Adox treatments were performed. Methyl inhibition decreased PDXK colocalization with ADMA and PRMT1 compared to dimethyl sulfoxide (DMSO) control treatments (fig. S4, A and B). Similarly, lysosomes marked by LAMP1 were also decreased by Adox conditions (fig. S4C). These data support a model whereby hyperactive lysosomal catabolism and Wnt activity maintain a continuous degradation of PDXK protein, which could be linked to the vitamin B_6_ deficiencies that are prevalent across patients with CRC.

### Vitamin B_6_ selectively determines CRC proliferation

**Fig. 4.**
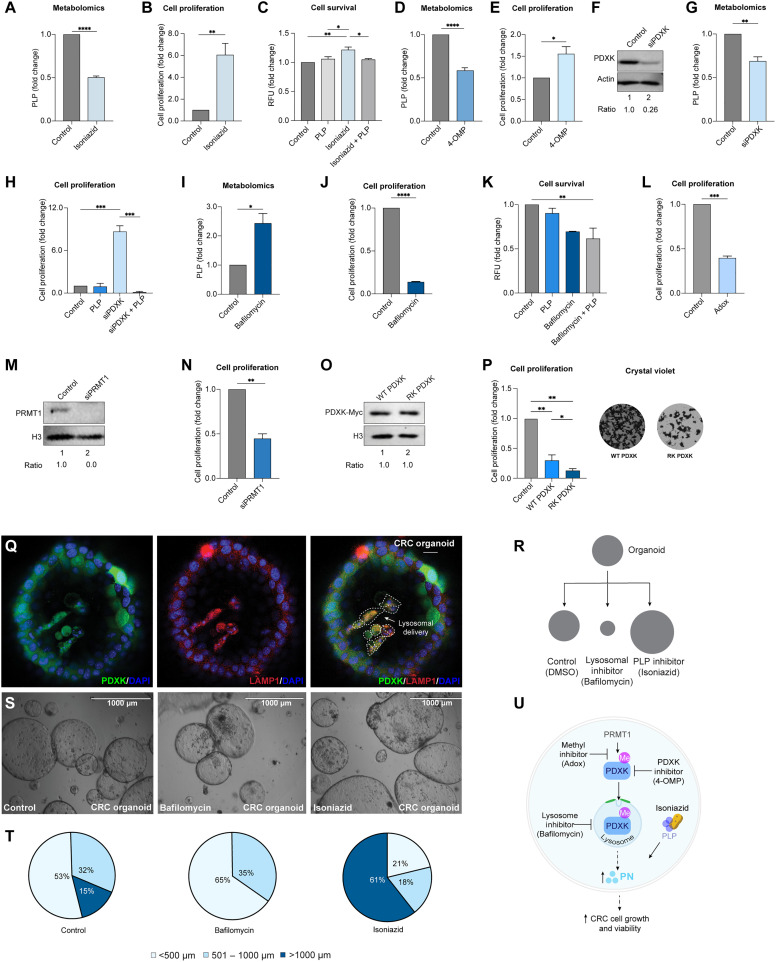
Disrupting PDXK in lysosomes restores B_6_ and reduces cell growth. (**A**) Intracellular PLP with isoniazid (300 μM) or dimethyl sulfoxide (DMSO) (5 days) assessed via GC-MS in SW480s. (**B**) Cell proliferation with isoniazid or DMSO. (**C**) Cell survival with isoniazid (24 hours) and PLP (24 hours) by PrestoBlue (fold change). (**D** and **E**) Intracellular PLP and cell proliferation with 4-OMP (100 μM). (**F**) PDXK with si*PDXK* or siScramble by IBs. (**G**) Intracellular PLP in si*PDXK* or siScramble cells. (**H**) Cell proliferation of si*PDXK* with a PLP rescue. (**I** and **J**) Intracellular PLP and cell proliferation following bafilomycin (200 nM) treatments. (**K**) Cell survival with bafilomycin and PLP (24 hours). (**L**) Cell proliferation with Adox (20 μM) or DMSO (48 hours). (**M**) si*PRMT1* or siScramble in SW480s by IBs. (**N**) Cell proliferation of si*PRMT1* or siScramble (5 days). (**O**) WT and RK PDXK expression in SW480s by IBs. (**P**) Cell proliferation and crystal violet staining in WT and RK PDXK expressing cells (5 days). (**Q**) Colocalization of PDXK (green) and LAMP1 (red) in intestinal organoids cultured from a genetic CRC mouse model (scale bar, 20 μm; 20×). Arrow indicates PDXK-LAMP1 colocalization. (**R**) Organoid growth assays include isoniazid, bafilomycin, or DMSO. (**S** and **T**) Organoids treated with bafilomycin, isoniazid, and DMSO (5 days) by EVOS imaging (scale bar, 1000 μm). Pie charts indicate organoid diameter quantification: isoniazid (average, >1000 μm), control (average, 380 μm), and bafilomycin (average, 184 μm). (**U**) Model of Wnt-PDXK-B_6_ axis in CRC. PDXK methylation and lysosomal degradation reduces PLP to promote growth. (A) to (Q) were performed in SW480s, and cell proliferation is shown after 5 days as fold change with DMSO controls. Data represent biological triplicates. *P < 0.05; **P < 0.01; ***P < 0.001; ****P < 0.0001. One-way ANOVA with Tukey’s correction in (C), (H), (J), and (L). Student’s *t* test in all other figures; means ± SEM. See also fig. S4.

We next aimed to understand the role of the Wnt-PDXK-B_6_ axis in CRC. To determine whether disrupting the Wnt-PDXK-B_6_ axis affected CRC, we first evaluated the effect of perturbing B_6_ metabolites on cell proliferation and survival. First, we leveraged isoniazid as a pharmacological approach to deplete intracellular PLP. Isoniazid is a Food and Drug Administration–approved small-molecule treatment for tuberculosis that has strong affinity for PLP and has been used as a method to deplete PLP for phenotypic cell- and tissue-based assays of B_6_ metabolism (*67*). SW480 cells were treated with isoniazid, and intracellular PLP levels were measured using high-performance gas chromatography–mass spectrometry (GC-MS) method. Peak intensities were confirmed by comparison with pure PLP standards. As anticipated, cells treated with isoniazid reduced intracellular PLP levels (Fig. 4A). Further, reduced intracellular PLP levels with isoniazid were accompanied by increased CRC proliferation (Fig. 4B and fig. S4E). To ensure that specificity of isoniazid effects was through B_6_, cell survival assays were performed, and the effects of isoniazid following the addition of PLP to cells were evaluated. Isoniazid increased cell survival compared to control DMSO treatments, while PLP addition was sufficient to reduce isoniazid effect to the level of control cells (Fig. 4C). To further investigate the contribution of PDXK, we used the PDXK-specific inhibitor, 4′-*O*-methylpyridoxine (4-OMP) (*68*). Similar to isoniazid, 4-OMP decreased intracellular PLP (Fig. 4D) and led to an increased rate of CRC proliferation (Fig. 4E and fig. S4E).

To confirm these pharmacological findings, a genetic knockdown approach was used with small interfering RNA (siRNA)–targeting PDXK. PDXK depletion in SW480 cells was confirmed in lysates by immunoblotting (Fig. 4F). Compared to scramble control, PDXK depletion was sufficient to decrease intracellular PLP (Fig. 4G), while PN levels increased (fig. S4F). Cell proliferation assays were performed in si*PDXK*-treated cells and decreased growth compared to control cells (Fig. 4H). To specifically confirm that the effects of PDXK depletion on cell proliferation were through B_6_, cell proliferation was assessed in PDXK-depleted conditions following PLP addition. PLP addition was sufficient to rescue the effects of PDXK depletion, as seen by the reduced rates of cell proliferation (Fig. 4H). Together, these data indicate that PDXK regulation of PLP plays an important role in CRC proliferation.

We next tested whether restoring PDXK protein levels would be sufficient to increase the levels of its product, PLP, and subsequently reduce growth of CRC. Toward this goal, we first examined the contribution of lysosomes to B_6_ metabolites and CRC proliferation. Cells treated with bafilomycin to inhibit lysosomes increased intracellular PLP levels (Fig. 4I) and decreased CRC cell growth by proliferation assays (Fig. 4J and fig. S4G). An additional lysosomal inhibitor, chloroquine, further showed a similar reduction in CRC proliferation (fig. S4G). To further confirm specificity, cell survival assays were performed with bafilomycin with or without PLP (Fig. 4K). Lysosomal inhibition decreased CRC cell survival compared to control DMSO treatments and had a combinatorial decrease in survival when combined with a PLP addition (Fig. 4J). These data suggest that lysosomal degradation of PDXK is an important regulator of B_6_ during cell proliferation.

We next aimed to test whether disrupting PDXK methylation via PRMT1 could offer an additional intervention point. Cell proliferation assays were first performed with PRMT inhibitors. As seen with lysosomal inhibition, CRC proliferation rates were reduced by PRMT inhibition with Adox, MS023, and GSK3368715 (Fig. 4L and fig. S4, H and I). To complement methylation inhibition studies, we next genetically depleted PRMT1 from SW480 cells using an siRNA approach. PRMT1 siRNA and siScramble confirmed PRMT1 depletion in lysates (Fig. 4M). As seen with the PRMT inhibitors, siRNA depletion of PRMT1 reduced cell proliferation compared to siScramble controls (Fig. 4N). To further examine the specific role of PDXK methylation, we next included a genetic approach using SW480 cells expressing WT or methyl-deficient RK PDXK. Immunoblotting was performed to confirm expression of WT and RK PDXK in SW480s (Fig. 4O). Compared to control cells, overexpression of WT PDXK reduced cell proliferation rates, and RK PDXK expression further exacerbated these effects on cell proliferation (Fig. 4P). Together, these genetic- and pharmacologic-based approaches support a model whereby PDXK methylation at the MrDegron motif leads to lysosomal degradation and reduces active B_6_ during CRC proliferation.

### PDXK is transported into lysosomes in colon organoids

To further evaluate this PDXK cytosolic-lysosomal shuttling process, we next turned to a three-dimensional organoid model derived from the small intestine of mice (*18*). Human CRC has been linked to multiple oncogenes and tumor suppressor proteins where 43% of patients have *Kras *mutations and 54% have a loss of *P53*. Therefore, we used a genetically engineered CRC mouse model containing the *Apc* knockdown,* KrasG12D *activation, and *P53* mutations (*37*, *69*) where small intestinal organoids have been shown to have multiple cell lineages and recapitulate organ physiological parameters. To start, we aimed to visualize PDXK in organoids and to evaluate whether PDXK localized into lysosomes. Cystic organoid morphology was consistent with previous reports, and lysosomes were found to be evenly distributed across all cell populations, including those within the internal organoid regions and around the periphery, as assessed by IF analyses (Fig. 4Q). In contrast, PDXK was primarily colocalized in LAMP1 within the internal organoid regions. Similar colocalization was observed for PDXK and ADMA in the internal organoid regions, in support of MrDegron targeting of PDXK into lysosomes (fig. S4J).

We next sought to functionally interfere with the PDXK proteolytic pathway to determine whether disrupting this axis could affect organoid growth rates. Organoids were treated with isoniazid to reduce PLP or with bafilomycin to inhibit lysosomes (Fig. 4R). Organoids retained a cystic morphology across all treatments, and diameters were measured. Compared to control treatments, isoniazid increased growth rates, which supports our findings of PLP depletion in colorectal cell lines (Fig. 4, S and T). In contrast, lysosomal inhibition with bafilomycin decreased organoid growth, even compared to controls. Together, these studies support a role for Wnt signaling and lysosomal metabolism in the regulation of B_6_, which may offer a potential vulnerability for growth in CRC (Fig. 4U).

## DISCUSSION

Vitamin B_6_ has recently emerged in the regulation of cellular metabolism in health and cancer. We report that canonical Wnt orchestrates dynamic fluxes of B_6_ vitamers during growth. Constitutive Wnt activity in *Apc* mutant cells displayed a continuous delivery of PDXK into lysosomes, which may explain previous reports that active B_6_ (PLP) is low in patients with CRC. MrDegron was found to be a protein tag that shunted PDXK into lysosomes and led to PN accumulation; genetic or pharmacologic disruption of the MrDegron molecular machinery blocked degradation and restored PLP, which offered multiple targets to reduce cell proliferation in CRC. Together, this work highlights the importance of PN and PLP vitamers in growth processes and provides mechanistic insight into the impact of vitamin levels in cancer.

### Wnt signaling and proteostasis

The present findings with canonical Wnt contribute to the emerging fields of research devoted to untangling how cells sense nutrient status and orchestrate protein catabolism. It has been well-described that before the transcriptional activity of β-catenin, Wnt ligands rapidly remodel cellular proteostasis. First, it was found that Wnt signaling reduces proteasomal degradation to stabilize protein levels during G_2_ before cell division, which has been attributed to the Wnt-mediated stabilization of proteins (Wnt/STOP). GSK3 catalyzes phospho-degrons for proteasomal degradation on 30% of the proteome, and GSK3 removal from the cytosol by Wnt increases total protein levels (*31*). Subsequently, an array of cytosolic targets beyond GSK3 were also found to be recruited into the lysosomes by Wnt ligands and included MrDegron substrates (*30*). Our findings support both models where Wnt affects proteasomes and lysosomes. We determined that during Wnt treatments, lysosomes were the primary mode of PDXK degradation, while in steady-state cells, both inhibitors of the proteasome and the lysosome stabilized PDXK. Wnt largely positioned PDXK for lysosomes, supporting a model whereby proteasomes are reduced by Wnt (*31*, *54*). Wnt increases lysosomal catabolism and restores free amino acids, which likely contribute to building macromolecules during tissue expansion (*33*). Why the cell would choose one proteolytic pathway over another remains an open question. Nevertheless, inhibitors of PRMTs or lysosomes restored PDXK levels and decreased cell growth in this study. Further, several PRMT inhibitors have made it to phase 1 trials for treatment of blood cancers. Thus, these findings highlight how targeting lysosomal pathways should be considered in cancer therapeutics.

### MrDegron and cytosolic catabolism in lysosomes

The current work highlights an emerging function of lysosomal targeting via MrDegron modifications. Protein methylation of lysine and arginine residues induce proteasomal degradation of several proteins (*62*, *70*). In contrast, MrDegron tags deliver proteins into lysosomes and target short-lived proteins for degradation (*30*). MrDegron modifications coordinated PDXK entry into lysosomes and modulated growth rates in a PLP-dependent manner. These findings highlight how time scales are integrated across biological systems; specifically, how growth factor signal transduction modulates metabolite abundance to a level that drives tissue remodeling. Posttranslational modifications via enzymatic activities (minutes) offer a link for integrating transcriptional activity (hours) and metabolite dynamics (seconds). Notably, the recompartmentalization of metabolic enzymes into distinct regions of the cell also enables metabolic, regulatory functions of the Wnt pathway that extend beyond GSK3 and the classic Wnt players.

### Wnt signaling, B_6_, and cancer cell metabolism

Wnt promotes Warburg metabolism and angiogenesis through pyruvate dehydrogenase kinase 1 (*Pdk1*) and lactate transporter (*Mct1*) expression (*20*). In CRC, lowering glutamine and α-ketoglutarate levels increased Wnt signaling, DNA hypermethylation, and cellular dedifferentiation (*21*). Our findings support a model whereby nongenetic metabolic factors contribute to tumorigenesis and include PLP as a Wnt target. Several lines of evidence suggest that bioactive B_6_ decreases oncogenesis and tumor progression in specific cancers. First, reduced circulating B_6_ levels have been correlated with worse survival in numerous cancers, while elevated circulating B_6_ reduces incidence of several distinct neoplasms (*71*). In addition, high intratumoral PDXK expression was associated with improved disease outcome. We report that Wnt shifted the balance of B_6_ vitamers to the inactive form PN and that continual PDXK degradation may explain the low levels of PLP (active B_6_) in human CRC (*41*, *49*). While our analyses of Wnt mutations and B_6_ levels focused on correlations in gastrointestinal tumors, low B_6_ has similarly been reported in numerous cancers known to display aberrant Wnt activity. Exciting progress has been made in cancer research wherein identifying the Wnt pathway step responsible for driving aberrant activity offers approaches for designing precise interventions (*72*, *73*). From a simplistic view, hyperactivity of β-catenin can occur outside of the cell—at the level of ligand secretion and Wnt receptor availability—or inside of the cell—through cytosolic regulators like the destruction complex. Beyond *β-catenin* mutations, recent reports describe how genetic mapping can lead to therapeutics by restabilizing the destruction complexes such as in cancers with APC-truncating mutations (*37*, *74*). Alternatively, Wnt receptor levels are regulated by two transmembrane E3 polyubiquitin ligases, ring finger protein 43 (RNF43) and zinc finger protein 3 (ZNRF3) (*72*, *73*). Loss-of-function mutations in *Rnf43* and *Znrf3* are frequently found in cancer cells (*72*). Upstream of this, additional players regulate RNF43-ZNRF3 E3 ligases where R-Spondin binds to leucine-rich repeat-containing G protein–coupled receptor (Lgr) and RNF43/ZNRF3 and trigger endocytosis and removal from the membrane (*72*, *73*). Conversely, the ubiquitin-specific peptidase 42 (USP42) protects ZNRF3 in the ternary complex of R-spondin-Lgr-ZNRF3 from auto-ubiquitination and clearance from the plasma membrane, counteracting this sophisticated control feedback (*72*, *73*). Together, genetic screening in patients could open avenues to use Wnt mutations as an index to decipher which tumors would be the most susceptible to B_6_ interventions.

### B_6_ deficiency, genetic mutations in PDXK, and isoniazid

PDXK is a key cofactor for enzymes in central metabolic pathways and contributes to human diseases (*9*, *11*). Despite the central role of PDXK across human physiology, the regulation of PDXK is only just emerging. This has been in part due to studies of isoniazid, a first-line tuberculosis treatment that has been used for over 70 years through bacterial growth inhibition via the suppression of enoyl-acyl carrier protein reductase, mycolic acids, and mycobacterial cell wall synthesis (*75*). Beyond this, isoniazid also depletes cellular PLP and has contributed to global understanding of B_6_ metabolism. For example, it was only through the use of routine isoniazid treatments that heterozygous PDXK mutations were identified because these patients suffered extreme B_6_ deficiency (*76*). Isoniazid treatments also showed that B_6_ deficiency increases risk for numerous cancers (*76*). In hepatocellular carcinoma, downstream targets of isoniazid reduction of B_6_ included interleukin-1B, cyclic adenosine monophosphate, and mammalian target of rapamycin (*76*). The antiproliferative effects of B_6_ have been attributed to redox balance control and to genomic stability (*3*). PDXK has antioxidant activities that counteract genotoxic molecules like reactive oxygen species and advanced glycation end products (*3*, *76*). Conversely, PDXK contributes to the progression of acute myeloid leukemia and clear cell renal carcinoma (ccRCC) (*77*, *78*). In ccRCC, the repression of urea cycle enzymes, arginase 2 and argininosuccinate synthase 1, promotes tumor growth in part by conserving PLP (*77*). Given this, genetic and metabolic dissection of these cancers relative to those where B_6_ is protective could help to elucidate the specific mechanisms through which PDXK tunes tumorigenic processes. Future work will focus on the genetic screening of PDXK mutations that could have diagnostic potential for elucidating patient populations that could be helped by restoring B_6_ through intervention at lysosomes or methyl-driven proteolysis.

## MATERIALS AND METHODS

### Cell lines and tissue culture

HeLa [American Type Culture Collection (ATCC), catalog no. CCL-2, RRID:CVCL_0030], HEK293T (ATCC, catalog no. CRL-1573, RRID:CVCL_0045), SW480 (ATCC, catalog no. CCL-28, RRID:CVCL_0546), SW620 (ATCC, catalog no. CCL-227, RRID:CVCL_0547), HCT116 (ATCC, catalog no. CCL-247, RRID:CVCL_0291), HT-29 (ATCC, catalog no. HTB-38, RRID:CVCL_0320), and L cells (ATCC, catalog no. CRL-2648) were cultured in Dulbecco’s modified Eagle’s medium (DMEM) with 10% fetal bovine serum (FBS), 1% penicillin-streptomycin, and 1% l-glutamine. Cells were grown at 37°C and 5% CO_2_.

### Immunofluorescence

Cells were grown in 10-cm dishes and transferred into 12-well dishes containing coverslips and left to adhere overnight. For HEK293T cells, coverslips were coated with poly-l-lysine (Sigma-Aldrich, P4707) before cell seeding. Endogenous Wnt3a secretion was inhibited using 5 μM IWP-2 (MilliporeSigma, I0536) for 1 hour following induction of Wnt signaling with Wnt3a ligands (PeproTech, 315-20) diluted 1:1000 in L cell (catalog no. CRL-2648) media for 20 min. For SW480 experiments, cells were treated with 20 μM Adox (Sigma-Aldrich, A7154) for 1 hour. Cells were washed with phosphate-buffered saline (PBS) to remove medium before cells were fixed with 4% paraformaldehyde (PFA) for 10 min. Next, cells were washed with PBS and permeabilized with 0.2% Triton X-100 for 10 min on ice. Cells were then washed with PBS before being blocked with 5% bovine serum albumin blocking buffer for 30 min, followed by incubation with primary antibodies (1:50 to 1:100, diluted in blocking buffer) overnight at 4°C. Last, cells were washed with PBS three times before incubation with secondary antibodies (1:5000, diluted blocking buffer) for 45 min and mounted onto glass slides in ProLong Gold antifade reagent with or without 4′,6-diamidino-2-phenylindole (DAPI, Invitrogen, P36931 and P36984) to stain cell nuclei. Images were acquired on Zeiss LSM-900 microscope with Airyscan using 20× and 40× water immersion and 63× oil immersion. The excitation lasers used to capture the images were 488, 568, and 405 nm using Alexa 568–, Alexa 488–, and Alexa 405–conjugated secondary antibodies. The same brightness/contrast profile was applied to all images within the same experiment. Ten images on 20×, 40×, and 63× were captured, and each image was considered a field. Zeiss and ImageJ imaging software were used for image analyses (10 fields per condition, at least 10 cells per field depending on magnification).

### Antibody information and reagents

The following antibodies were used for IF and immunoblotting analyses. PDXK antibodies were purchased from Sigma-Aldrich (HPA030196, RRID:AB_10599735) and Santa Cruz Biotechnology (sc-365173, RRID:AB_10708566). Asymmetric di-methyl arginine antibodies were purchased from Abcam (ab413, RRID:AB_304302) and Thermo Fisher Scientific (PA5-120706, RRID:AB_2914278). PRMT1 (SAB5701542, RRID:AB_2940956), β-actin (A1978, RRID:AB_476692), HA (SAB2702217, RRID:AB_2750919), and Flag (F1804, RRID:AB_262044) antibodies were purchased from Sigma-Aldrich. Glyceraldehyde-3-phosphate dehydrogenase (CST 2118, RRID:AB_561053), myc (CST 2272 RRID:AB_10692100), Histone H3 (CST 3638, RRID:AB_1642229), and LAMP1 (CST 58996, RRID:AB_2927691) were purchased from Cell Signaling Technology. Goat anti-rabbit IgG horseradish peroxidase (HRP, 31460, RRID:AB_228341), rabbit anti-mouse IgG secondary antibody HRP (61-6520, RRID:AB_2533933), goat anti-mouse Alexa Fluor 405 (A48255, RRID:AB_2890536), goat anti-rabbit Alexa Fluor 405 (A-31556, RRID:AB_221605), goat anti-mouse Alexa Fluor 488 (A28175, RRID:AB_2536161), goat anti-rabbit Alexa Fluor 488 (A-11008, RRID:AB_143165), and goat anti-rabbit Alexa Fluor 568 (A-11011, RRID:AB_143157) were purchased from Thermo Fisher Scientific.

### Cell proliferation and survival assays

Cells were seeded into 12-well dishes at 1 × 10^5^ cells/ml and treated in triplicates with 0.2 μM bafilomycin (Cayman Chemical, 11038), 20 μM Adox (Sigma-Aldrich, A7154), 300 μM isoniazid (MilliporeSigma, I3377), 4-OMP (BOC Sciences, 1464-33-1), 500 μM chloroquine (Sigma-Aldrich, C6628), 2 μM GSK3368715 (MedChemExpress, HY-128717A), and 10 μM MS023 (Sigma-Aldrich, SML1555) for 3 and 5 days. After 3 and 5 days, wells were trypsinized, resuspended in fresh media, and counted using the Countess II Automated Cell Counter. The fold changes in cell number were calculated on the basis of the seeding density, followed by normalization against the control, where the fold change for control was always set as 1.0. Fold changes of live cells in drug conditions were plotted against the control. For crystal violet staining, wells were fixed in 4% PFA, stained with 0.5% crystal violet (Sigma-Aldrich, C6158), and imaged using EVOS M5000 imaging system.

Cell survival was also measured using PrestoBlue Cell Viability Reagent (Invitrogen, A13261) according to the manufacturer’s instructions. Cells were grown in 96-well plates at 1.5 × 10^4^ cells per well before triplicate treatments with 0.02% DMSO, 20 nM bafilomycin, and 5 μM isoniazid for 24 hours. Following this initial incubation, PLP (10 μg/ml; Sigma-Aldrich, P3657) was added to wells and incubated for 24 hours to induce rescue. PrestoBlue was then added to wells and incubated for 3 hours at 37°C. Plates were read using a Synergy H1 BioTek fluorescence microplate reader at 560 nm excitation and 590 nm emission. The fold changes in the relative fluorescence unit were normalized and plotted against the control.

### Western blotting

Cell lysates were prepared by the addition of 2× Laemmli sample buffer [4% SDS, 20% glycerol, 120 mM tris-Cl (pH 6.8), 0.02% bromophenol blue, and 5% β-mercaptoethanol, resolved by 4 to 20% SDS–polyacrylamide gel electrophoresis (SDS-PAGE)] and boiling at 95°C for 10 min. Samples were resolved by 4 to 20% SDS-PAGE using standard protocols. Blocking buffer (5% milk in PBS) was used to block nitrocellulose membranes for 1 hour at room temperature shaking. All antibodies were diluted in blocking buffer and incubated overnight at 4°C with rocking, washed with PBS, and incubated with secondary antibodies for 1 hour at room temperature with shaking. Images were acquired using the iBright FL1500 imaging system. Bands were quantified using ImageJ software and normalized to the individual loading controls before being normalized to the control sample. Cycloheximide (Sigma-Aldrich, C1988) and MG132 (AdooQ Bioscience, A11043) were used at 10 and 5 μM, respectively. All other drug treatment concentrations were used as previously described in cell proliferation assays.

### Site-directed mutagenesis and DNA constructs

The full-length PDXK construct was purchased from Origene (RC200975). Mutation of R292 into lysine was carried out using the QuikChange site-directed mutagenesis kit (Agilent, 200523) according to the manufacturer’s instructions with the following 5′-to-3′ primers: TTTTGCTCTGCACCATCTTCAGCTCCAGCTGCATGG; CCATGCAGCTGGAGCTGAAGATGGTGCAGAGCAAAA. Mutagenesis was confirmed by DNA sequencing. HA-tagged TMEM192–red fluorescent protein (RFP) HEK293T (HA-Lyso) cells were obtained from R. Zoncu’s laboratory, as previously described (*57*). WT and DN VPS4 were previously described (*30*).

### Plasmid and siRNA transfections

Cells were grown in DMEM supplemented with 10% FBS, penicillin-streptomycin, and l-glutamine in 37°C and 5% CO_2_. When cells reached 50% confluency, HeLa and HEK293T cells were transiently transfected with WT and mutant *PDXK *constructs using Lipofectamine 2000 (Invitrogen, 11668019) according to the manufacturer’s instructions. SW480 cells were transfected with siRNAs using Lipofectamine RNAiMax (Invitrogen, 13778100) according to the manufacturer’s protocol. After 24 hours of transfection, medium was exchanged. Cell treatments varied on the basis of experiments but followed the same drug concentrations as previously described. Treatment was followed by either IF, Western blotting, metabolomics, or cell proliferation analyses. The ON-TARGETplus Human SMARTpool siRNA oligos for si*PDXK* (L-005070-00-0010), si*PRMT1* (L-010102-00-0005), and siScramble RNA were purchased from Dharmacon.

### Protease protection assay

Protease protection assays were performed to determine protein localization in membrane-bound organelles. For in situ protease protection assays, cells were incubated with Wnt3a ligands for 20 min, placed on ice, and washed with PBS. Cells were then treated with Digitonin (6.5 μg/ml; Sigma-Aldrich, D141) for 5 min, followed by a 10-min incubation with Proteinase K (1 μg/ml; Invitrogen, 100005393). Negative control wells were treated with Triton X-100 in addition to Proteinase K, digesting all membranes and allowing for Proteinase K to degrade all proteins. Reactions were stopped with 4% PFA and examined by IF assays described previously.

### PDXK IP and Lyso-IP

IP of PDXK-FLAG (Origene, RC200975) was performed in HEK293T cells. Cells were transiently transfected with PDXK-Flag using Lipofectamine 2000. The following day, cells were pretreated for 2 hours with bafilomycin (100 nM). During the final hour of pretreatment, cells were treated with IWP-2 (5 μM) in serum-free DMEM. Cells were then treated with Wnt3a protein (80 ng/μl) and bafilomycin (100 nM) for 1 hour and were harvested in lysis buffer [50 mM tris-HCl (pH 7.9), 150 mM NaCl, 1 mM EGTA, 1 mM EDTA, 10 μM fresh leupeptin, 100 μM fresh 4-(2-Aminoethyl)-benzenesulfonyl-fluoride (AEBSF), and 2 mM fresh phenylmethylsulfonyl fluoride]. Cells were centrifuged at 13,000 rpm for 5 min at 4°C. Samples were incubated with anti-FLAG M2 magnetic beads (Sigma-Aldrich, M88230) for 1.5 hours, rotating at 4°C. The samples were then washed once with tris-buffered saline, eluted in 2× Laemmli, and boiled. Clarified lysates and immunoprecipitated samples were then analyzed by SDS-PAGE and Western blot as described above. Blots were probed with anti-PDXK, -Myc, and -ADMA antibodies.

### Lysosome immunoprecipitation

HEK293T cells stably expressing HA-tagged TMEM192-RFP were used to immunoprecipitated lysosomes, as previously described (*57*, *58*). Briefly, cells were grown to 50 to 75% confluency and treated with Wnt3a ligands as previously described. For overexpression experiments, HA-tagged TMEM192-RFP HEK293T cells were transfected with WT and mutant *PDXK *before Wnt3a treatment. Cells were washed and scraped with PBS followed by centrifugation. Cells were resuspended in KPBS buffer [136 mM KCl, 10 mM KH_2_PO_4_, 0.5 mM tris(2-carboxyethyl)phosphine (TCEP), and Opti-Prep protease inhibitor cocktail, (pH 7.25)] and lysed with 23G syringes followed by another round of centrifugation. Fifty microliters of the post-nuclear supernatant (PNS) was set aside and resuspended in 2× Laemmli buffer for immunoblotting analysis (lysate). The rest of the PNS was incubated with PBS-washed anti-HA beads (Thermo Fisher Scientific, 88836) and rotated at 4°C for 1 hour. Anti-HA beads were then centrifuged, and pellets were resuspended in Laemmli buffer for immunoblotting analysis. Last, anti-HA beads were washed in KBPS buffer three times for 5 min rotating at 4°C, eluted in 2× Laemmli, and boiled. Clarified lysates and immunoprecipitated samples were then analyzed by SDS-PAGE and Western blot as described above. Similarly, HA-tagged TMEM192-RFP HEK293T cells overexpressing WT and mutant Flag-tagged PDXK were immunoprecipitated using anti-FLAG M2 beads, as described above. Blots were probed with anti-PDXK, -HA, and -myc antibodies.

### Gas chromatography–mass spectrometry

For HeLa metabolomics, cells were seeded at 2 × 10^5^ cells per well in six-well plates in DMEM supplemented with 10% FBS and 1% penicillin-streptomycin in an incubator with 37°C and 5% CO_2_. Cells were treated with Wnt3a ligand for 20 and 60 min. Extraction began by placing the six-well dishes on ice and washing each well with 2 ml of a 150 mM ammonium acetate solution (pH 7.4, 4°C). After wash, metabolites were extracted from each well on dry ice with 500 ml of a (1:4) dH_2_O, MeOH solution at 80°C. The samples were then incubated for 15 min at 80°C. Afterward, the metabolite solutions were transferred to Eppendorf tubes and spun down at 4°C for 10 min at 17,000*g*. The supernatants were transferred to fresh vials and dried with an EZ2-Elite lyophilizer (Genevac). Dried metabolites were resuspended in 100 ml (1:1) of dH_2_O:acetonitrile (ACN) solution. After resuspension, metabolite solutions were spun at 17,000*g* for 10 min. The top 70 ml of the supernatant was then transferred to high-performance liquid chromatography (HPLC) autosampler vials. Ten milliliters of these suspensions was injected per analysis. Samples were run on a Vanquish (Thermo Fisher Scientific) UHPLC system with mobile phase A [5 mM NH_4_AcO (pH 9.9)] and mobile phase B (ACN) at a flow rate of 200 ml/min on a Luna 3-mm NH_2_ 100A (150 × 2.0 mm) at 40°C with a gradient going from 15 to 95% A in 18 min followed by an 11-min isocratic step. The UHPLC was coupled to a Q-Exactive (Thermo Fisher Scientific) mass analyzer running in polarity switching mode at 3.5 kV with an MS1 resolution of 70,000. Metabolites were identified via exact mass (MS1), retention time, and in some cases by their fragmentation patterns (MS2) at normalized collision energy. Quantification was performed via area under the curve integration of MS1 ion chromatograms with the MZmine 2 software package. Area values were then normalized to cell count averages from triplicate wells treated in parallel for each condition.

For metabolomics, SW480 cells were seeded at 2 × 10^5^ cells per well in six-well plates in DMEM supplemented with 10% FBS and 1% penicillin-streptomycin in an incubator with 37°C and 5% CO_2_. Cells were treated in triplicates with bafilomycin (200 nM), isoniazid (300 μM), and Adox (20 μM) for 5 days. After treatment, cells were washed with cold saline and put on dry ice. One milliliter of 80% methanol/water (HPLC grade) with norvaline as internal standard was added to cells followed by transferring the plates to a −80°C freezer for 15 min to inactivate enzymes. The whole-cell extract was collected and transferred to a tube then centrifuged at 17,000*g* for 10 min at 4°C. The supernatant was dried by the speed vacuum and incubated with 50 μl of methoxyamine hydrochloride (MOX) (10 mg/ml in pyridine) at 42°C for 1 hour. After cooling down, the samples were incubated with 100 μl of tert-Butyldimethylsilyl ether (TBDMS) at 70°C for 1 hour. PLP and PN (Sigma-Aldrich, P5669) were used as standards. Samples were analyzed by Agilent 7820A gas chromatograph system and Agilent 5977 mass spectrometer.

### Enzymatic digestion and LC-MS/MS analysis

For the identification of R292 methylation, 5 μg of GST-PRMT1 (Cayman Chemical, 10350) and 5 μg of His-PDXK (R&D Systems, 8658-PK) were incubated in the presence or absence of 200 μM SAM (Sigma-Aldrich, A4377) in 30 μl of reaction buffer [50 mM tris-HCI (pH 8.0), 20 mM KCI, 5 mM dithiothreitol (DTT), and 4 mM EDTA], and incubated at 37°C for 1 hour. PRMT1 was removed from the reactions using glutathione resin (Sigma-Aldrich, G0924, room temperature, 30 min). The remaining PDXK was reduced with DTT (8 mM, 60°C, 15 min) and alkylated (50 mM iodoacetamide, room temperature, dark, 45 min) before digestion with sequencing grade trypsin [Promega, 1/50 (w/w), 37°C, 16 hours]. Samples were desalted on C18 ZipTips (Millipore) and analyzed by matrix-assisted laser desorption/ionization–time-of-flight MS (Bruker Ultraflextreme). Observed *m*/*z* were mapped to an in silico digestion of PDXK using Biopharmalynx (Waters Corp, v 1.3.5, 30 parts per million, fixed carbamidomethyl Cys, variable oxidation of Met, variable Arg methylation). To confirm peptide assignments, aliquots of each tryptic digest were analyzed by LC-MS using an ACQUITY UPC H-class LC coupled to an Xevo Q-ToF (Waters Corp). Peptides were injected onto a reverse phase C4 column (ACQUITY UPLC Protein BEH C4 Column, 300 Å, 1.7 μm, 2.1 mm by 50 mm, Waters Corp) in a buffer stream of 3% ACN in 0.1% formic acid (0.3 ml/min) and desalted for 0.5 m before gradient elution from 3 to 60% ACN over 1.5 m. The Xevo Z-spray source was operated with a capillary voltage of 3000 V and a cone voltage of 40 V (NaCsi calibration, Leu-enkephalin lock-mass). Nitrogen was used as the desolvation gas at 350°C and a total flow of 800 liter/hour.

### Organoid culture and growth assay

Small intestinal* shApc/Kras/P53^−/−^* organoids were provided by the Lukas Dow laboratory (Weill Cornell Medicine) through the Kong laboratory (UC Irvine) and grown based on established protocols (*21*). In brief, organoids were grown in IntestiCult Organoid Growth Medium (STEMCELL Technologies, 06005) supplemented with primocin, mixed 1:1 with growth factor–reduced Matrigel (Corning, 356230), plated onto 12-well plates, and left to polymerize at 37°C. Once polymerized, 750 μl of intestinal organoid media was added to cover the Matrigel dome. Organoid medium was changed every other day and split 1:5 every week. For passaging, organoid medium was aspirated, and the Matrigel was dissociated using EDTA-PBS for 10 min, with shaking, at room temperature.

For the organoid growth assay, organoids (day 3 after seeding) were treated in triplicates with 0.1% DMSO, 0.2 μM bafilomycin, or 1.6 mM isoniazid for 5 days. Three 20× images were taken for each condition (*n* > 10 organoids per image). Organoid diameter was measured in micrometers based on EVOS M5000 imaging system scale bar. Organoid diameters were divided into three ranges (<50, 51 to 1000, and >1000 μm). Numbers of organoid diameters in each range per experimental condition were graphed.

### Organoid staining and confocal imaging

Organoids were treated with 0.2 μM bafilomycin and 20 μM Adox for 1 hour. Organoids were washed with PBS and fixed with 4% PFA in 4°C. Matrigel was dissolved in PFA, and organoids were loosened by gentle shaking and transferred into falcon tubes followed by three PBS washes. Organoids were transferred into eight-well glass-bottom chamber slides and treated with blocking buffer (5% horse serum and 0.5% Triton X-100 in 1× PBS) at 4°C overnight. Organoids were incubated in primary antibodies (1:50, diluted in blocking buffer) at 4°C overnight. Organoids were washed three times with 1× PBS for 10 to 15 min and stained with secondary antibody (1:5000, diluted in blocking buffer without Triton X-100) at 4°C overnight. Last, organoids were washed three times in 1× PBS for 10 to 15 min and stained with DAPI at 5 μg/ml in 1× PBS for 5 min at room temperature, followed by a final three washes in 1× PBS for 10 to 15 min. Images were acquired on a Zeiss LSM-900 microscope with Airyscan using 2.5×, 10×, and 20× magnifications. The excitation lasers used to capture the images were 488, 568, and 405 nm (DAPI) using Alexa 568– and Alexa 488–conjugated secondary antibodies. Zeiss and ImageJ imaging software were used for image analyses.

### Proximity ligation assay

Reagents for the PLA of HeLa cells were purchased and used using instructions from Sigma-Aldrich (DUO92004 and DUO92002). Briefly, cells treated with Wnt ligands for 20 min, fixed on coverslips, and prepared for staining with primary antibodies as described above. Anti-PDXK, anti-LAMP1, and anti-IgG were used at 1:100 dilutions and anti-ADMA was used at 1:50 dilutions. Anti-IgG was used as a negative control. Instead of fluorescently tagged secondary antibodies, specimens were incubated with anti-mouse and anti-rabbit oligonucleotide-tethered secondary antibodies. Close proximity of target antigens allows their respective secondary antibody nucleotide sequences to hybridize. Following a 30-min 37°C incubation with ligase and additional oligonucleotides, a closed DNA circle is formed. A subsequent step involving polymerase-driven rolling circle amplification incorporates fluorescently labeled nucleotides. Fluorescent nucleotides used in this experiment fluoresce in the red channel upon excitation. A fluorescent spot appears at sites of PDXK-LAMP1 or PDXK-ADMA protein-protein interactions using primary antibodies and were evaluated using confocal microscopy and quantified using ImageJ [National Institutes of Health (NIH), http://imagej.nih.gov/ij/]. Data were normalized by applying the same brightness/contrast profile and threshold values. ImageJ’s analyze particles feature was used to quantify PLA spots over 10 fields of view for each condition (*n* > 30). Raw values for each paired experiment (PDXK-LAMP1, PDXK-ADMA, and PDXK-IgG) were then converted to fold change with respect to the appropriate control.

### In vitro methylation assay

Methylation assays in vitro were performed on the basis of previous reports (*79*). In short, 5 μg of His-PDXK (R&D Systems, no. 8658-PK) and 200 μM SAM (Sigma-Aldrich, no. A4377) were incubated in the presence or absence of 5 μg of GST-PRMT1 (Cayman Chemical, no.10350) in 30 μl of reaction buffer [50 mM Tris-HCl (pH 8.0), 20 mM KCl, 5 mM DTT, and 4 mM EDTA], and incubated at 37°C for 1 hour. The mixture was then boiled with 2× Laemmli sample buffer and subjected to immunoblotting analysis with anti-ADMA (1:500 dilution), anti-PDXK (1:5000), and anti-PRMT1 (1:1500) antibodies.

### PDXK structural model

Human PDXK sequence (O00764) was obtained from UniProt (https://uniprot.org) and input into BLAST (NIH Basic Local Alignment Search Tool, https://blast.ncbi.nlm.nih.gov/Blast.cgi) as a query sequence. All sequences not having 100% query coverage and 100% percent identity (97 sequences) were downloaded and aligned using Clustal Omega (https://ebi.ac.uk/Tools/msa/clustalo/). Aligned sequences were then input into the ConSurf server (https://consurf.tau.ac.il/consurf_index.php) to produce a three-dimensional evolutionary conservation model of PDXK.

### Statistical analysis

All experiments were repeated independently with similar results three times unless otherwise specified. All data represent means ± SEM. GraphPad Prism 9 software was used for statistical analyses. Two-tailed Student’s *t* test was used to compare variables between two groups. One-way analysis of variance with Tukey’s correction was performed to compare three or more groups against each other. *P < 0.05, **P < 0.01, ***P < 0.001, and ****P < 0.0001 were considered to be statistically significant, while ns indicated nonsignificance. Image quantification was performed as described previously.
