# Supplementary material for: Vitamin B6 is governed by the local compartmentalization of metabolic enzymes during growth
Source: Sci Adv. 2023 Sep 8;9(36):eadi2232. doi: 10.1126/sciadv.adi2232 (PMC10491294; doi:10.1126/sciadv.adi2232)
Supplement: Supplementary file 1 — Figs. S1 to S4 [file sciadv.adi2232_sm.pdf]

Science Advances
AAAS

Supplementary Materials for

Vitamin B_6 is governed by the local compartmentalization of metabolic enzymes during growth

Carolina N. Franco *et al.*

Corresponding author: Lauren V. Albrecht, l.albrecht@uci.edu

Sci. Adv. **9**, eadi2232 (2023)
DOI: 10.1126/sciadv.adi2232

This PDF file includes:

Figs. S1 to S4

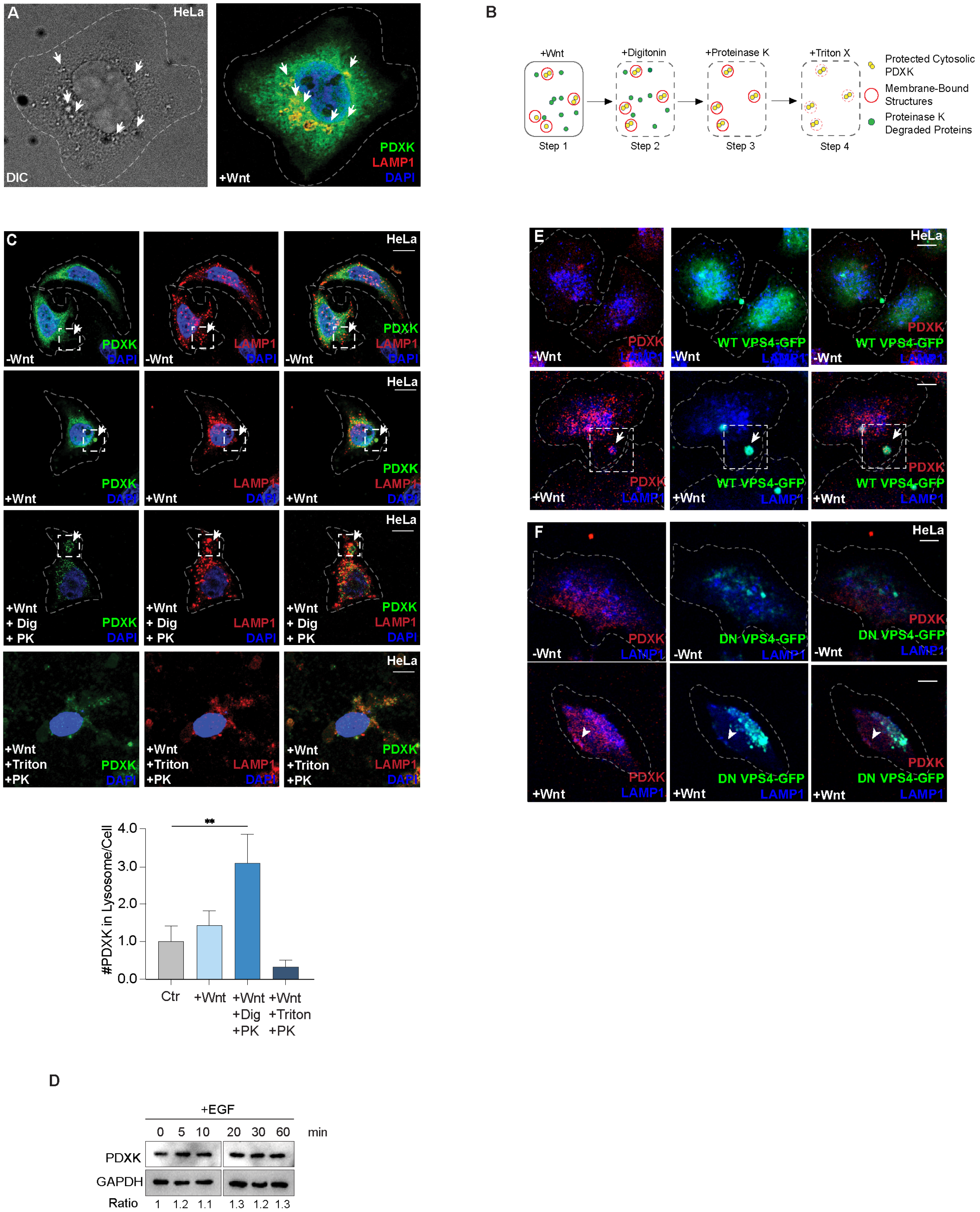

Fig. S1. PDXK is Delivered into Vesicular LAMP1 Structures with Functional VPS4
(**A**) Endogenous PDXK (green) and LAMP1 (red) in HeLa cells treated with Wnt or control ligands by IF and DIC analyses. Arrows indicate PDXK colocalization into punctate lysosomal structures. (**B**) Scheme of protease protection assay. Live cells are treated with digitonin, which targets cholesterol patches that are only found within the plasma membrane and leaves

intracellular organelles intact. Proteinase K degrades cellular contents that are not contained within membrane-bound structures. Positive control treatments to confirm protein localization in membrane-bound structures include Triton X, which permeabilizes all membranes. (**C**) Endogenous PDXK (green) and LAMP1 (red) staining in control or Wnt treated cells by IF. PDXK and LAMP1 continue to colocalize in Wnt-treated cells following digitonin permeabilization and proteinase K. Arrows indicate PDXK protected in lysosomes. Triton X permeabilization of all membranes exposes PDXK to proteinase K and leads to its degradation. Quantification was performed using 10 fields ($n < 10$) at 20X magnification. (**D**) PDXK protein levels following EGF time course by IBs. Ratios represent levels of PDXK relative to loading control. (**E and F**) Endogenous PDXK colocalization with LAMP1 in WT VPS4-GFP or DN VPS4-GFP expressing HeLa cells during Wnt or control treatments (20 minutes). Scale bars, 10 µm. All data represent biological triplicates. *$P < 0.05$; **$P < 0.01$; ***$P < 0.001$; ****$P < 0.0001$. Student's t-test; mean ± SEM.

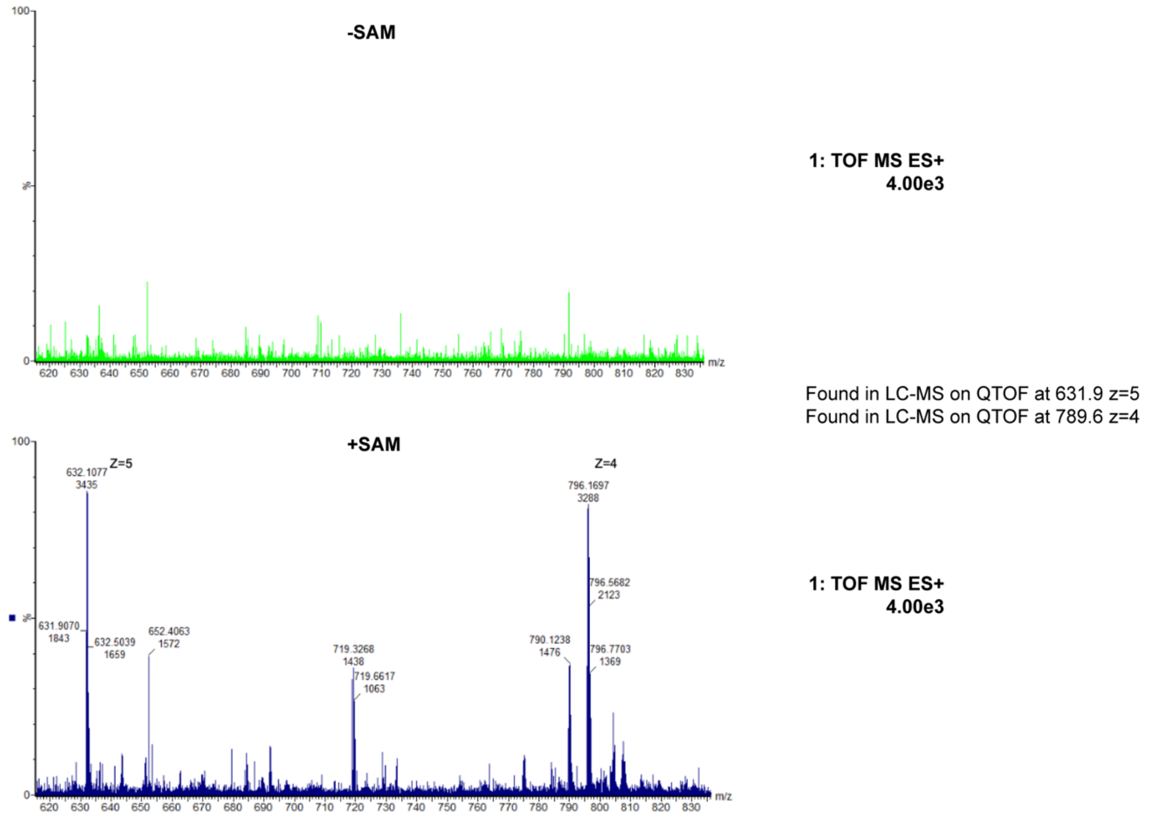

**PDXK**	TIQCAKAQAGEGVRPSPMQLELRMVQSK	1:T028-030*	Methyl x2 R(1),Carbamidomethyl C(1),Methyl R(1)	2.8	5	631.9036	3154.4783
**PDXK**	TIQCAKAQAGEGVRPSPMQLELRMVQSK	1:T028-030*	Methyl x2 R(1),Carbamidomethyl C(1),Methyl R(1)	2.8	4	789.6107	3154.4111

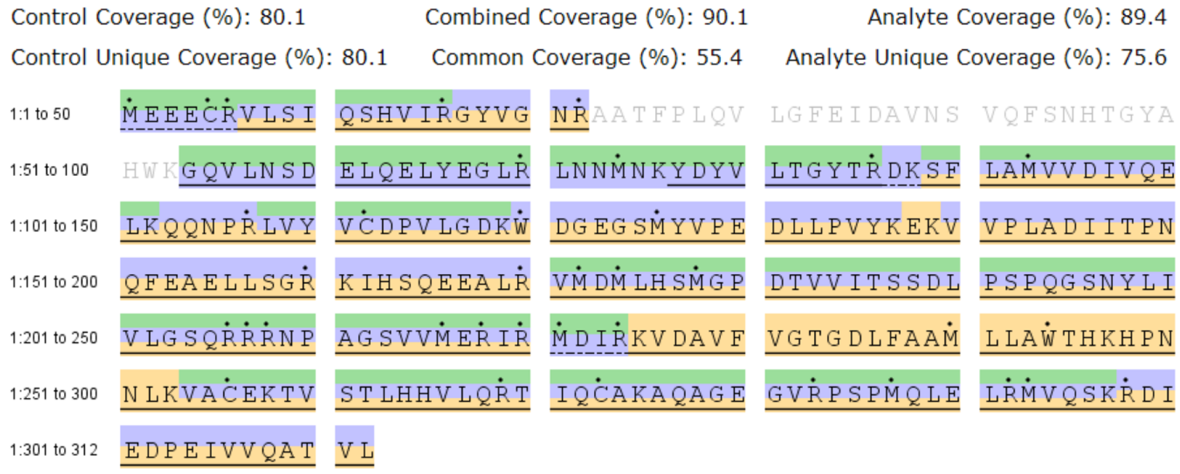

Fig. S2. PDXK is Modified by Arginine Methylation at Residue 292
Mass spectrometry was performed using in vitro methylated PDXK samples from recombinant PDXK and PRMT1 together with S-Adenosyl Methionine (SAM). PDXK Arg292 methylation is

confirmed by LC-MS. Protein coverage of at least 90.1% was observed in the MS analyses of PDXK. An m/z of 3155.6, consistent with the modified peptide r.TIQCAKAQAGEGVRPSPMQLELRMVQSK.r (270T-297K, carbamidomethyl Cys, di-methyl Arg, methyl Arg), was found only in the +SAM MALDI spectrum. The target peptide was observed in the LC-MS analysis (+SAM condition only) in multiple charge states with an average error of 41 ppm.

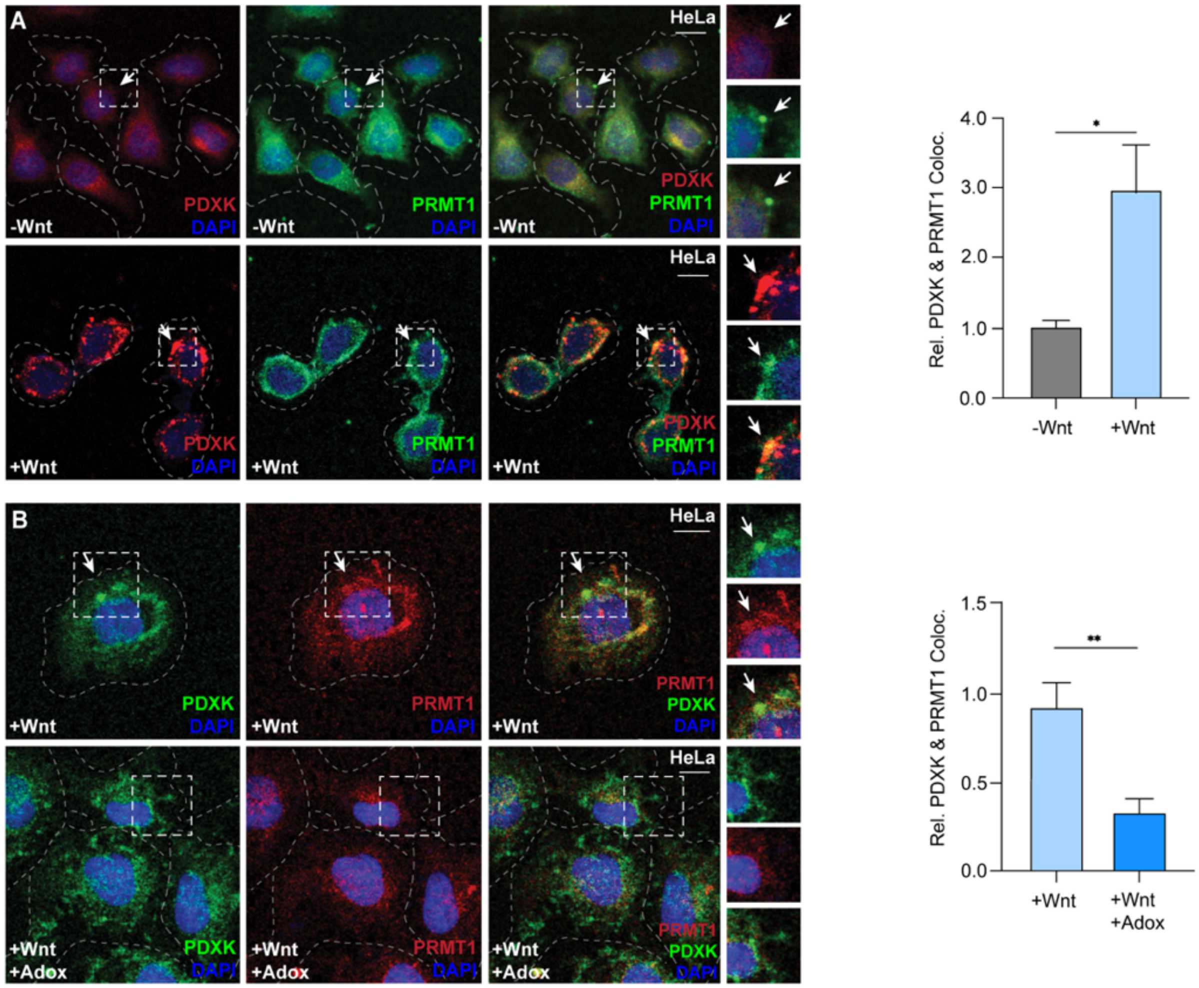

Fig. S3. Wnt Signaling Induces PDXK colocalization with PRMT1
(**A**) PDXK and PRMT1 in Wnt or control treatments for 20 minutes in Hela cells, assessed by immunofluorescence (IF) (Scale bars indicate 10 μm). Displayed images were taken at 20X magnification. Quantification of PDXK and PRMT1 colocalization induced by Wnt treatments was performed using 10 fields (n < 80). (**B**) PDXK and PRMT1 colocalization with methylation inhibitor (Adox) or DMSO during Wnt treatments for 20 minutes in Hela cells (Scale bars indicate 10 μm at 20X magnification). Quantification of PDXK and PRMT1 was assessed over 10 fields (n < 80). All data represent biological triplicates. *P < 0.05; **P < 0.01; ***P < 0.001; ****P < 0.0001. Student's t-test; mean ± SEM.

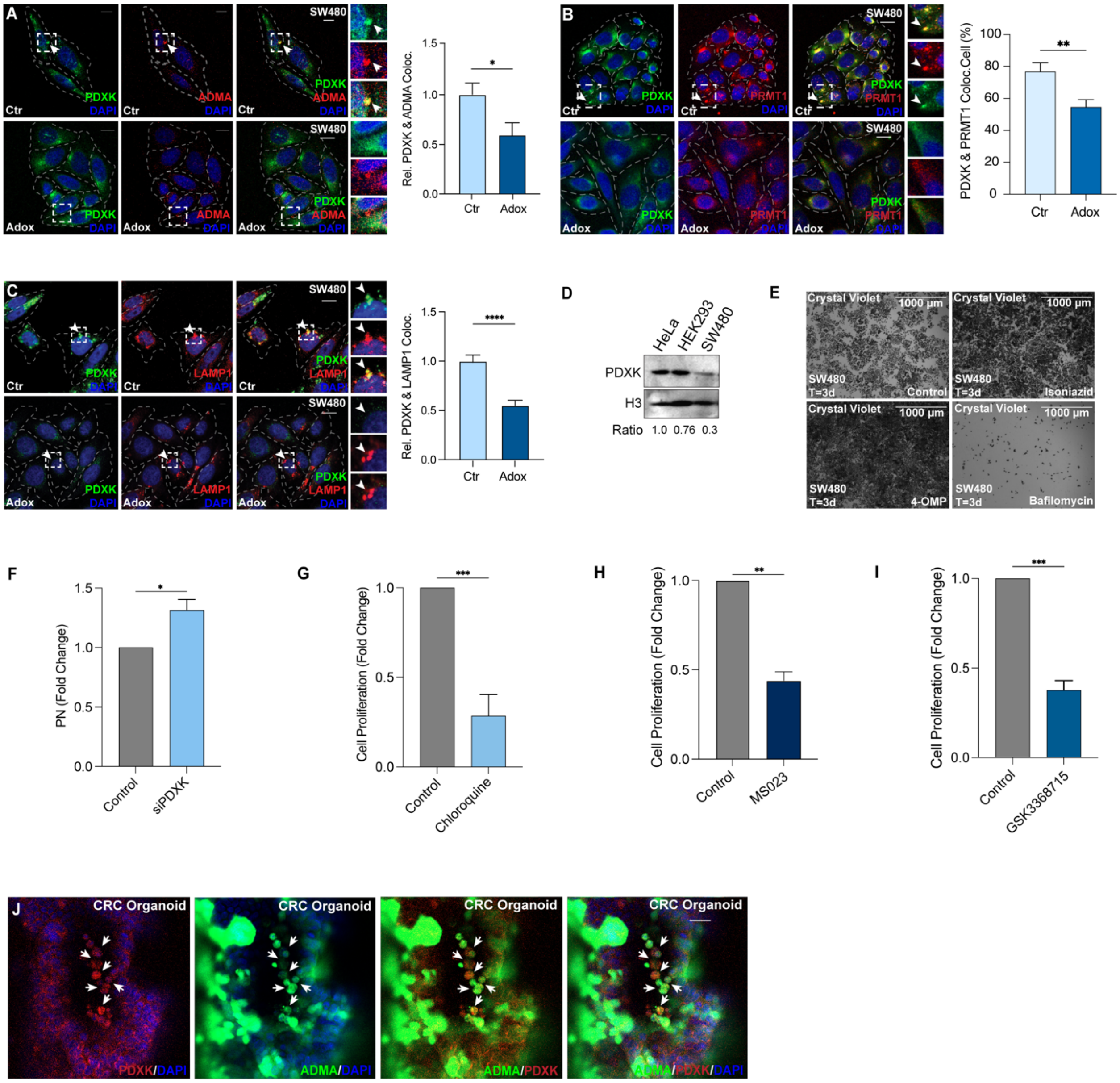

Fig. S4. PDXK is Localized to Lysosomes with PRMT1 and ADMA in CRC
(**A**) PDXK (green) and ADMA (red) in SW480 cells following treatment with protein methylation inhibitor (Adox) or DMSO by IF. Arrows indicate PDXK and ADMA colocalization. 20X magnification. Quantification was performed using 10 fields (n < 50) at 20X magnification. Scale bars, 10 µm. (**B**) PDXK (red) and PRMT1 (green) in SW480 cells by IF. Arrows indicate PDXK colocalization with PRMT1. 20X magnification. Scale bars, 10 µm. Quantification was performed using 10 fields (n < 50) at 20X magnification. (**C**) PDXK (green) and LAMP1 (red) in SW480s with Adox or DMSO treatments by IF. Arrows indicate PDXK colocalization with lysosomes. 20X magnification. Quantification was performed using 10 fields (n < 50) at 20X magnification. Scale bars, 10 µm. (**D**) Endogenous PDXK levels in HeLa, HEK293T, and SW480 cells analyzed by IBs. (**E**) Crystal violet staining of viable SW480 cells following treatments with DMSO, isoniazid, 4-OMP, or bafilomycin after 3 days. Imaged using EVOS M5000 imaging system. 4X magnification. Scale bars, 1000 µm. (**F**) Cellular PN levels in

siPDXK SW480 cells after 5 days measured via GC/MS using a PN standard. (**G**) Fold changes in cell proliferation of SW480 cells following treatments with chloroquine (500 μM) after 2 days. (**H**) Fold changes in cell proliferation of SW480 cells following treatments with MS023 (10 μM) after 3 days. (**I**) Fold changes in cell proliferation of SW480 cells following treatments with GSK3368715 (2 μM) after 4 days. (**J**) PDXK colocalizes with ADMA in CRC organoids. 20X magnification. Scale bars, 20 μm. All data represent biological triplicates. $^{*}P < 0.05$; $^{**}P < 0.01$; $^{***}P < 0.001$; $^{****}P < 0.0001$. One-way ANOVA with Tukey's correction in (G). Student's t-test in all other figures; mean ± SEM.
